# Detection of hypervirulence genes in carbapenem resistant *Klebsiella pneumoniae* from cancer patients at a tertiary referral hospital in Nepal

**DOI:** 10.1186/s12879-026-13382-8

**Published:** 2026-04-23

**Authors:** Archana Katuwal Chhetri, Hari Prasad Dhakal, Banita Gurung, Sanjib Adhikari, Supriya Sharma, Megha Raj Banjara, Komal Raj Rijal, Prakash Ghimire

**Affiliations:** 1 Central Department of Microbiology, Tribhuvan University, Kirtipur, Nepal; 2 Nepal Cancer Hospital and Research Center (NCHRC), Lalitpur, Nepal

**Keywords:** Carbapenem resistance, Hypervirulent gene, *K. pneumoniae*, Cancer, *bla*_NDM−1_, *iucA*

## Abstract

**Background:**

Carbapenem-resistant *Klebsiella pneumoniae* (CRKP) are a serious public health threat and is linked to high mortality, particularly in immunocompromised and critically ill patients. In addition, hyper-virulent *K. pneumoniae* (hvKP) variants contributing to severe clinical outcomes in cancer patients. This study aims to identify carbapenem-resistant *K. pneumoniae* isolates from cancer patients and detect associated hypervirulence genes.

**Methods:**

Clinical specimens were collected from cancer patients and processed for bacterial isolation and identification by conventional method. The antibiotic susceptibility testing was performed using Kirby Bauer disk diffusion method following CLSI guideline. All carbapenem-resistant *K. pneumoniae* isolates were further analyzed for carbapenemase production using the modified carbapenem inactivation method (mCIM) and metallo-β-lactamase (MBL) production using the EDTA-modified carbapenem inactivation method (eCIM). The presence of carbapenemase and hypervirulence encoding genes in CRKP were confirmed by polymerase chain reaction (PCR). All data were analyzed using SPSS. The chi-square test was applied to assess associations, and a p-value < 0.05 was considered statistically significant at a 95% confidence interval.

**Results:**

Out of 4273 samples, bacterial growth was observed in 23% (987/4273) with a total of 1016 bacterial isolates, including polymicrobial growth. A total of 23.7% (241/1016) of *K. pneumoniae* isolates were retrieved from 241 cancer patients. Carbapenem-resistant *K. pneumoniae* (CRKP) isolates were detect in 53.5% (129/241), with 38% (49/129) of *bla*_NDM−1_ as predominant carbapenemase gene. Among hypervirulence markers, *iucA* (66.7%, 86/129) was the most prevalent. Co-occurrence of carbapenem resistance genes and hypervirulence genes was detected in 21% (27/129) among CRKP.

**Conclusion:**

Effective intervention strategies are crucial to enhance the timely clinical recognition and optimal management of CRKP and hvKP infections.

**Supplementary Information:**

The online version contains supplementary material available at 10.1186/s12879-026-13382-8.

## Introduction

*Klebsiella pneumoniae* is an opportunistic pathogen responsible for a wide range of community- and hospital-acquired infections [1]. The rapid emergence of antimicrobial resistance (AMR) has made it a major global health concern. The World Health Organization has listed *K. pneumoniae* as a high-priority pathogen due to its increasing resistance to multiple antibiotics [2].

Antimicrobial resistance in *K. pneumoniae* is mediated by mechanisms such as enzymatic inactivation, efflux pump overexpression, and porin loss. Among these, carbapenemase production is particularly concerning, as it confers resistance to last-resort β-lactam antibiotics, including carbapenems [3, 4]. As a result, carbapenem-resistant *K. pneumoniae* (CRKP) has emerged as a critical global threat [5]. Common carbapenemases include Ambler class A (KPC), class B (NDM, VIM, IMP), and class D (OXA-48-like enzymes), which are frequently associated with severe hospital infections [6].

Traditionally, *K. pneumoniae* strains have been classified into two pathotypes: classical *K. pneumoniae* (cKp) and hypervirulent *K. pneumoniae* (hvKp). Classical strains are typically associated with healthcare settings and antimicrobial resistance, whereas hvKp strains are generally more virulent and often susceptible to antibiotics [7]. Unlike classical cKP, which is mainly linked to hospital-acquired infections, hvKP is more commonly seen in community settings and is known for causing more severe and invasive diseases, including pneumonia, liver abscess, meningitis, and necrotizing fasciitis [8]. Hypervirulent strains are characterized by enhanced virulence factors, including a hypermucoviscous phenotype regulated by *rmpA* and/or *rmpA2*, and increased iron acquisition mediated by siderophores such as aerobactin (*iucABCD-iutA*), salmochelin (*iroBCDEN*), yersiniabactin, and enterobactin [9]. These features enable hvKp to cause severe, invasive infections, even in otherwise healthy individuals [10].

Recently, the convergence of resistance and virulence has led to the emergence of two hybrid forms: carbapenem-resistant hypervirulent *K. pneumoniae* (CR-hvKP), referring to *hvKp* strains that acquire carbapenem resistance, and hypervirulent carbapenem-resistant *K. pneumoniae* (hv-CRKP), referring to *CRKP* strains that acquire hypervirulence determinants. This convergence is primarily driven by horizontal gene transfer via mobile genetic elements [11–13].

These hybrid strains are increasingly reported worldwide and are associated with severe clinical outcomes. Studies from Asia indicate a rising prevalence of CR-hvKP, highlighting the growing overlap between resistance and virulence traits [14–16].

Cancer patients are particularly susceptible to infections due to immunosuppression, frequent hospital exposure, and the use of invasive procedures. In this population, *K. pneumoniae* infections have been reported approximately 10–18% of cases, highlighting its significant role as a causative pathogen among patients with malignancies [17–19]. Moreover, infections caused by multidrug-resistant and hypervirulent *K. pneumoniae* are associated with increased morbidity, mortality, and limited therapeutic options [20]. Notably, mortality rates among cancer patients with *K. pneumoniae* infections remain high, ranging from 40% to 72.7% [21, 22], underscoring the severe clinical impact of these resistant strains.

In Nepal, data on the coexistence of carbapenem resistance and hypervirulence in *K. pneumoniae*, especially among cancer patients, remain limited. Therefore, this study aimed to detect and characterize carbapenem-resistance and hypervirulence-associated genes in *K. pneumoniae* isolates from cancer patients, addressing an important knowledge gap.

## Materials and methods

### Study design

A hospital-based cross-sectional study was carried out at the Nepal Cancer Hospital and Research Center (NCHRC) between June 2023 and May 2024. Ethical approval (ref no: 113–2023) was obtained from Ethical Review Board, National Health Research Council (NHRC). All cancer patients who visited the NCHRC, and provided written informed consent during the study period were enrolled as study participants. During this period, a total of 4,273 clinical specimens were analyzed from 3,504 cancer patients after excluding duplicate samples. To ensure data accuracy, repeat specimens from the same patient were only included if they were collected during readmission for a new episode of treatment. Molecular analysis was performed at the Central Department of Microbiology.

### Specimen collection, bacterial culture and identification

Inclusion criteria: The clinical samples from cancer patients (both inpatient and outpatient) suspected of infections were collected by trained medical personnel and received in the Microbiology Laboratory of Pathology Department. Samples from the same patients when re-admitted for next episode of the diagnosis and treatment were included for analysis.

Exclusion criteria: Duplicate samples for the same person for same period, samples without proper label and contaminated/spilled samples were excluded.

All specimens including pus, wound swab, body fluid, sputum, tips, blood and urine were processed as soon as possible according to standard microbiological procedures for bacterial isolation and phenotypic identification [23]. MacConkey agar and blood agar were used for culturing pus, wound swabs, aspirates, and catheter tips. Chocolate agar was additionally used for sputum samples, while cystine–lactose–electrolyte-deficient (CLED) agar was used for urine cultures. Sterility of culture media was ensured by incubating 5% of randomly selected prepared media aerobically at 37 °C for 24 h. Quality control of the media was performed using reference strains of *Staphylococcus aureus* (ATCC 25923) and *Escherichia coli* (ATCC 25922).

### Antibiotic susceptibility testing

Antibiotic susceptibility testing was performed by Kirby-Bauer disk diffusion technique following the guidelines of CLSI. The antibiotics disks used were ampicillin/clavulanic acid (20/10 µg), piperacillin/tazobactam (100/10 µg), ceftazidime (30 µg), cefotaxime (30 µg), cefoxitin (30 µg) cefepime (5 ug), levofloxacin (5 µg), ciprofloxacin (5 µg), norfloxacin (10 µg), imipenem (10 µg), meropenem (10 µg), doripenem (10 µg), gentamycin (30 ug), tobramycin (10 µg), amikacin (30 µg), tigecycline (15 µg), doxycycline (30 µg), tetracycline (30 µg), aztreonam (30 ug), nitrofurantoin (300 µg) and colistin (10 µg) (HiMedia India Pvt. Ltd., Bengaluru, India).The inoculum was prepared by picking parts of similar test organisms with a sterile wire loop and suspended in sterile normal saline. To standardize the density of the inoculum of bacterial suspension, 0.5 McFarland turbidity standard was used. The test organism was uniformly seeded over the Mueller-Hinton agar surface and exposed to a concentration gradient of antibiotic diffusing from antibiotic-impregnated paper disk into the agar medium and incubated at 37 °C for 16 to 18 h. Diameters of the zone of inhibition around the discs were measured to the nearest millimeter using a ruler and classified as sensitive, intermediate and resistant. Note: Colistin susceptibility was interpreted with caution and, where applicable, confirmed using broth microdilution as recommended [24].

### Phenotypic screening and confirmation for carbapenem resistance

Carbapenemase production was initially screened by the disk diffusion method. Isolates showing reduced susceptibility to meropenem or imipenem (inhibition zone ≤ 22 mm) were considered potential carbapenemase producers. Phenotypic detection was performed using the modified Carbapenem Inactivation Method (mCIM) and EDTA-modified CIM (eCIM) following CLSI guidelines. Briefly, a loopful of overnight culture of *K pneumoniae* was inoculated into two tubes containing trypticase soya broth, one with EDTA, 0.5 M, 10 µL (eCIM) and one without (mCIM). A meropenem (10 µg) disc was added to each tube and incubated at 35 °C for 4 h. The discs were then placed on Mueller–Hinton agar plates inoculated with *Escherichia coli* ATCC 29,522 and incubated at 35 °C for 18–24 h. An inhibition zone diameter < 18 mm indicated carbapenemase production, while ≥ 19 mm indicated a negative result. An increase of ≥ 5 mm in the inhibition zone diameter in eCIM compared to mCIM was interpreted as metallo-β-lactamase (MBL) production [24]. Bacteria non-susceptible to at least one agent in three or more antimicrobial categories were considered as multidrug-resistant (MDR) [25].

#### String test 

The isolates were incubated overnight on blood agar plates at 37 °C. Fresh colonies from overnight cultures were then gently stretched using an inoculation loop. The formation of visible mucous filaments with a stretching length greater than 5 mm was interpreted as a positive result, indicating a hypermucoviscous phenotype [26].

### Detection of carbapenemase and virulence encoding genes

Genomic DNA from carbapenem-resistant isolates was extracted using a Qiagen DNA extraction kit. In addition, carbapenem resistance genes were identified by targeting carbapenemase-encoding genes (*bla*_IMP−1_, *bla*_VIM−2_, *bla*_NDM−1_, *bla*_KPC_, and *bla*_OXA−48-like)_. Polymerase chain reaction (PCR) was performed to detect virulence-associated genes, including *iucA*,* rmpA*,* rmpA2*,* iroB*, and *peg-344*. PCR amplification was carried out with an initial denaturation at 95 °C for 2 min, followed by 35 cycles of denaturation at 95 °C for 20 s, annealing at 52 °C for 40 s, and extension at 72 °C for 1 min, with a final extension step at 72 °C for 5 min. Amplified PCR products were resolved by agarose gel electrophoresis and visualized under ultraviolet illumination. Band sizes were determined by comparison with a 100-bp DNA ladder and corresponding positive controls. A negative control containing all PCR reagents except template DNA was included in each run [27–29]. Primer sequences used in this study are provided in Supplementary Table 1.

### Statistical analysis

Data were entered and analyzed using SPSS v 21 and Chi-square tests and fisher exact test were used to find out association of string test with hypervirulence encoding gene, association of hypervirulence encoding and carbapenemase encoding gene, and association of clinical history and treatment status with CRKP infection. A p-value < 0.05 was considered statistically significant. Venn diagrams were created using R software version 4.2.

## Results

### Baseline demographic, clinical, and microbiological characteristics of the study population

The bacterial growth of clinical significance was observed in 23% (987/4273) of the samples, single bacterial growth was found in 97% (958/987) samples and mixed bacterial growth was found in 3% (29/987) samples with a total of 1016 bacterial isolates, retrieved from 851 cancer patients.

A total of 23.7% (241/1016) of *K. pneumoniae* isolates were recovered from 241 cancer patients. Of these, 50.6% (122/241) were from male patients and 49.4% (119/241) from female patients. The patients’ ages ranged from 2 to 91 years, with most belonging to the ≥ 61 years age group. The majority of isolates (94.2%) were obtained from patients with solid malignancies. Prior antibiotic use was reported in 52% (125/241) of total patients. Hospitalization for more than 3 days was observed in 38.5% (93/241) of patients, while 11.6% (28/241) were from ICU admission. The majority of patients (77.6%, 187/241) were undergoing chemotherapy at the time of assessment. Fever was the most common clinical feature, present in 68% (164/241) of cases (Table 1).

**Table 1:** Demographic and clinical characteristics of cancer patients

Variable	*n* (%)
**Gender**	
Male	119(49.4)
Female	122(50.6)
**Age(years)**	
≤ 15	5(2)
16–45	36(15)
46–60	74(30.7)
≥ 61	126(52.3)
**Type of cancer**	
Solid	227(94.2)
Hematologic	14(5.8)
**Prior antibiotic use**	
Yes	125(52)
No	116(48)
**Hospitalization (> 3days)**	
Yes	93 (38.5)
No	148(61.5)
**ICU admission**	
Yes	28(11.6)
No	213(88.4)
**Chemotherapy status**	
On treatment	187(77.6)
Not on treatment	54(22.4)
**Fever**	
Yes	164(68)
No	77(32)

### Distribution of *K. pneumoniae* in clinical specimens

Among total *K. pneumoniae* isolates, 42% were obtained from urine specimen, followed by sputum (25.3%), catheter tips (10.4%), body fluids (7%), wound swabs (6.2%), pus (5,4%) and blood (3.7%) (Table 2).

**Table 2:** Distribution of *K. pneumoniae* isolates among different samples

Bacterial			Type of sample				
Isolates	Fluid	Blood	tips	pus	Sputum	urine	wound swab
	n (%)	n (%)	n (%)	n (%)	n (%)	n (%)	n (%)
*K. pneumoniae*	17 (7)	9 (3.7)	25 (10.4)	13 (5.4)	61 (25.3)	101 (42)	15 (6.2)

### Antibiotic resistance in *K. pneumoniae* isolates

*K. pneumoniae* isolates showed 100% susceptibility to colistin. Low resistance was observed to tigecycline (9.5%) and doxycycline (32.8%). In contrast, high resistance was observed to amoxicillin–clavulanate (99.6%) and cefoxitin (79.7%). For carbapenems, 53.5% of isolates were resistant to both imipenem and meropenem (Table 3).

**Table 3:** Antibiotic resistance in *K. pneumoniae* isolates

Antimicrobial category	Antibiotics	Resistant	
		No. of isolates (*n* = 241)	%
Aminoglycosides	Amikacin	115	47.7
	Gentamycin	122	50.6
	Tobramycin	138	57.3
Beta lactam combination	Piperacillin Tazobactam	151	62.7
	Amoxycillin Clavulanic acid	240	99.6
Monobactam	Aztreonam	129	53.5
Fluroquinolones	Ciprofloxacin	156	64.7
	Levofloxacin	156	64.7
	Norfloxacin(*N* = 101)*	74	73.3
Cephalosporins	Cefoxitin	192	79.7
	Ceftazidime	180	74.7
	Cefotaxime	181	75.1
	Cefepime	162	67.2
Carbapenem	Meropenem	129	53.5
	Imipenem	129	53.5
	Doripenem	130	53.9
Tetracycline	Tetracycline	127	52.7
	Doxycycline	79	32.8
Nitrofuran	Nitrofurantoin (*N* = 101)*	75	74.3
Glycylcycline	Tigecycline	23	9.5
Polymyxin	Colistin	0	0.0

*Nitrofurantoin and Norfloxacin were used only for isolates from urine samples

### Phenotypic and genotypic detection of carbapenem-resistant *K. pneumoniae* (CRKP)

**Fig. 1:**
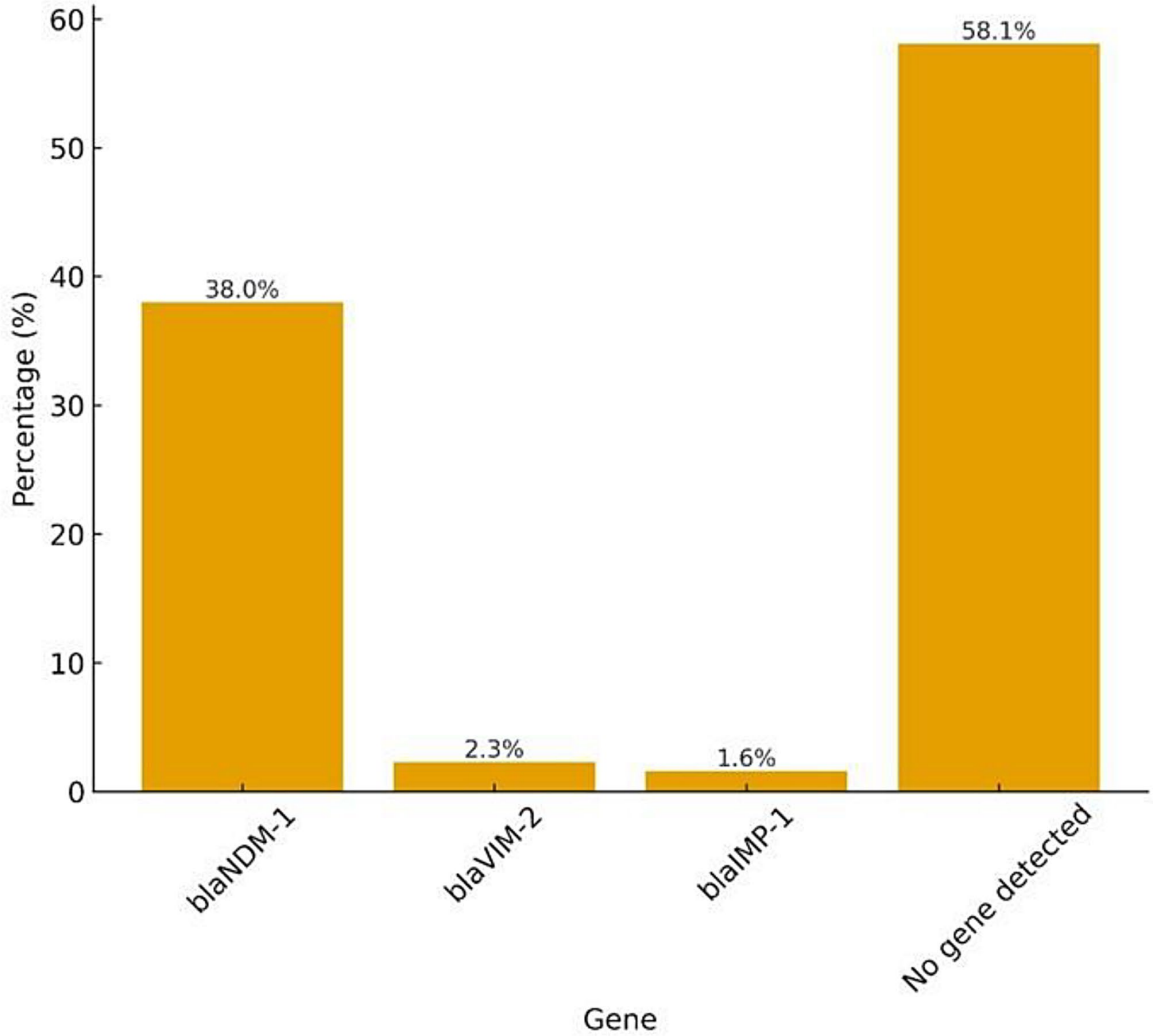
Distribution of carbapenemase encoding gene

Among 241 *K. pneumoniae* isolates, 71% (171/241) of isolates were multidrug-resistant (MDR), 53.5% (129/241) of the isolates were carbapenem-resistant (CRKP) and 43% (104/241) of the total isolates were MBL producer. Molecular analysis showed, 41.8% (54/129) of the CRKP isolates harbored carbapenemase gene. The *bla*_NDM−1_ gene was the most prevalent, detected in 38% (49/129) of the isolates, followed by *bla*_VIM−2_ 2.3% (3/129) and *bla*_IMP−1_ 1.6% (2/129) (Fig. 1).

### Genotypic and phenotypic identification of hvKp

Hypervirulence-associated genes were detected in 76% (98/129) of carbapenem-resistant *K. pneumoniae* (CRKP) isolates. Among the investigated virulence biomarkers, *iucA* was the most prevalent gene, identified in 66.7% (86/129) of isolates followed by *rmpA2* (34%), *iroB* (27%), *peg-344* (10%), and *rmpA* (2.3%) (Table 4).

**Table 4:** Detection of hypervirulence encoding gene (*N* = 129)

Hypervirulence genes	Number	Percentage
*iucA*	86	66.7
*iroB*	35	27
*peg344*	13	10
*rmpA*	3	2.3
*rmpA2*	44	34
total	181*	

*Note: The total (181) is the cumulative detection of genes, not the total number of isolates, since multiple genes can coexist in a single isolate

The string test was performed on all isolates of CRKP isolates. Among the 47string test positive CRKP isolates, 76.6% (36/47) carried hypervirulence genes. The hypervirulence genes were detected in 75.6% (62/82) among string test negative isolates. Statistically, significant association was not observed between string test positivity and the presence of hypervirulence gene carrying isolates (*p* > 0.05), indicating that hypermucoviscosity alone is not only marker for hypervirulence (Table 5).

**Table 5:** Association of string test and hypervirulence-associated genes among CRKP isolates

Hypervirulence genes (*iucA, iroB, peg344, rmpA* and* rmpA*2)	String test positive *n* (%)	String test negative *n* (%)	*p* value
Presence	36(76.6)	62(75.6)	0.8996
Absence	11 (23.4)	20(24.4)	
Total	47	82	

### Co- occurrence of carbapenemase and hypervirulence encoding gene

**Fig. 2:**
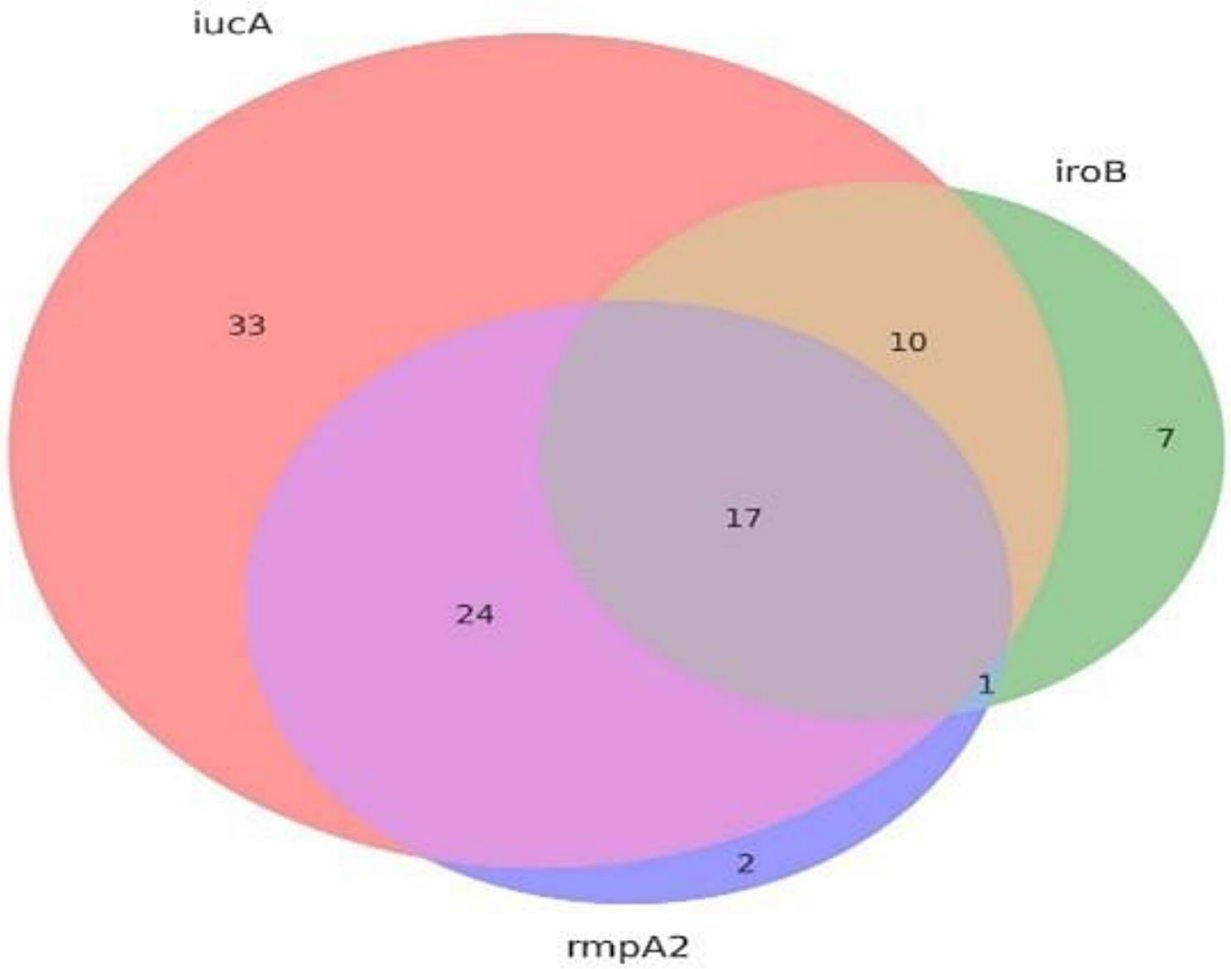
A Venn diagram showing the distribution and overlap of the hypervirulence-associated genes *iucA*, *iroB*, and *rmpA2* among *K. pneumoniae* isolates. The diagram illustrates the proportion of isolates carrying individual genes as well as their combinations, with *iucA* being the most frequently detected

**Fig. 3:**
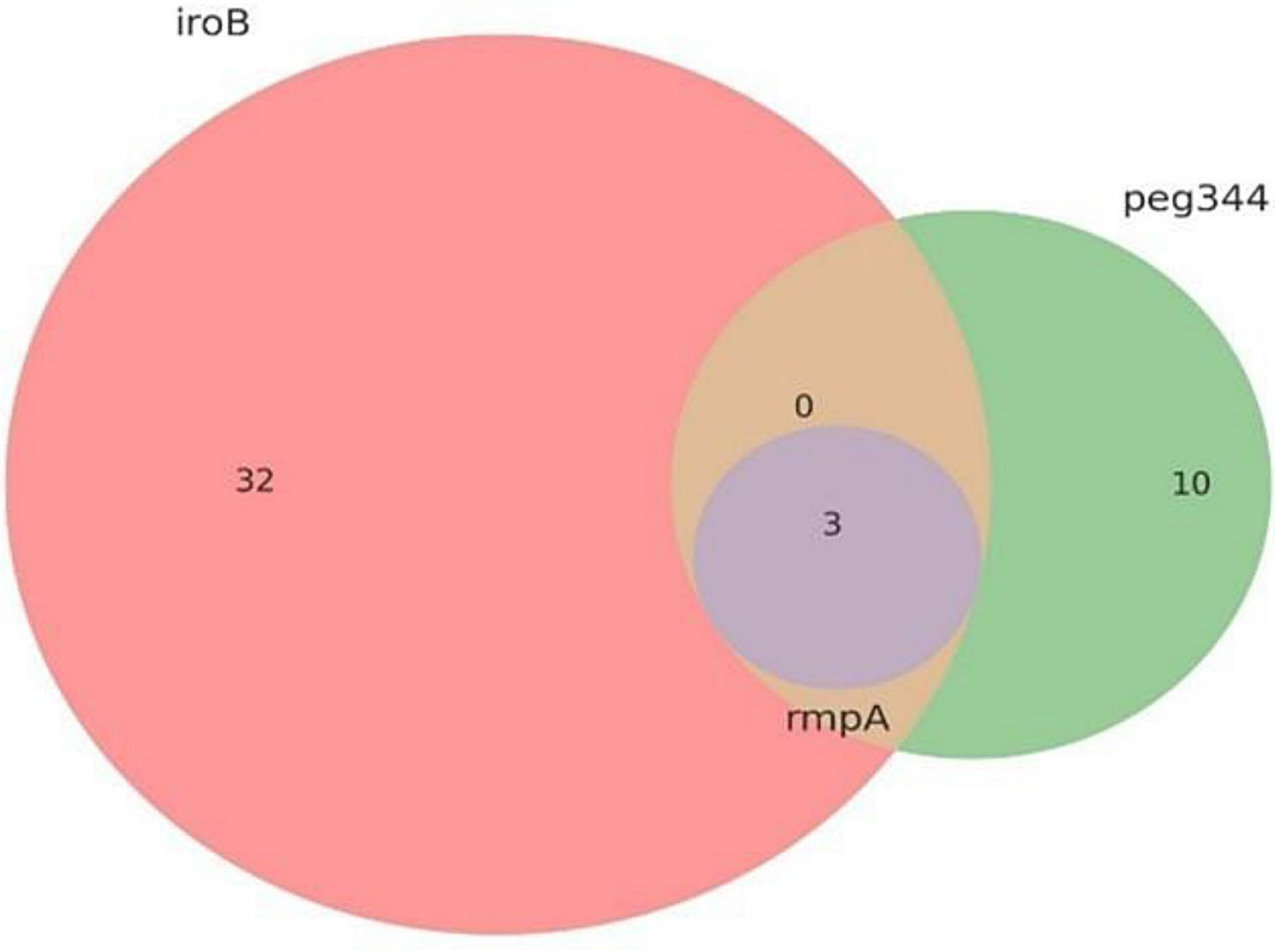
Venn diagram illustrating the distribution and overlap of the hypervirulence-associated genes *iroB*, *peg344*, and *rmpA* among *K. pneumoniae* isolates. The majority of isolates carried *iroB* alone, while *peg344* and *rmpA* were less frequently detected. A small subset of isolates harbored all three genes concurrently, indicating the presence of highly virulent genetic profiles

A total of 21 distinct gene-combination patterns were identified among 129 *K. pneumoniae* isolates. The most common pattern was bla_NDM−1_ alone 19.4% (25/129), indicating a high prevalence of carbapenem resistance. Virulence genes were frequently detected, with *iucA* alone 11.6% (15/129) and *iucA* with *rmpA2* 14.0% (18/129) being the predominant virulence combinations. Notably, the co-existence of carbapenemase-encoding resistance genes and hypervirulence-associated genes was observed in 21% (27/129) among CRKP isolates which termed as carbapenem resistant hypervirulent *K. pneumonia*e (CR-hvKP). The most frequent combination was bla_NDM−1_ with *iucA*, detected in 11.6% (15/129) of isolates. Less common combinations included bla_NDM−1_ with *iroB* in 4.6% (6/129) and bla_NDM−1_ with rmpA2, in 1.5% (2/129) of isolates. Additionally, single isolates (0.8%; 1/129 each) harbored bla_VIM−2_ with *iucA*, bla_NDM−1_ with *iucA* and *iroB*, bla_VIM−2_ with peg-344, and bla_IMP−1_ with *iucA* (Figs. 2 and 3).

Gene combinations in carbapenem resistant *K. pneumoniae* are provided in supplementary Table 2.

### Association of carbapenemase encoding gene and hypervirulence genes

Among carbapenemase producing isolates, *iucA* was detected more frequently (33%, 18/54) than in carbapenemase non-producing isolates (9.3%, 7/75), showing a significant association. (*p* < 0.001). In contrast, *iroB* was rare among carbapenemase producing isolates (4%, 2/54) but common in carbapenemase non-producing isolates (44%, 33/75). Similarly, *peg-344* and *rmpA2* were significantly less prevalent in carbapenemase producing isolates compared with non-producing isolates, showing a significant association. The *rmpA* gene was not detected in any carbapenemase producer (Table 6).

**Table 6:** Association between carbapenemase and hypervirulence encoding genes

SN	Hypervirulence encoding genes		carbapenemase encoding genes (*bla*_NDM−1_, *bla*_VIM−2_, *bla*_IMP−1_) (*N* = 54)		
			Positive	Negative	
1	*iucA* (*N* = 86)	Positive	18 (33)	68 (91)	< 0.001
		Negative	36 (67)	7 (9)	
2	*iroB* (*N* = 35)	Positive	2 (4)	33 (44)	< 0.001
		Negative	52 (96)	42 (56)	
3	*peg-344* (*N* = 13)	Positive	1 (2)	12 (16)	0.008
		Negative	53 (98)	63 (84)	
4	*rmpA* (*N* = 3)	Positive	0 (0.0)	3 (4)	0.264
		Negative	54 (100)	72 (96)	
5	*rmpA2* (*N* = 44)	Positive	2 (4)	42 (56)	< 0.001
		Negative	52 (96)	33 (44)	

### Association of clinical history and treatment status with CRKP infection

Prior antibiotic use, prolonged hospitalization (> 3 days), ICU admission, and presence of fever were significantly more common among patients with CRKP infections compared to non-CRKP infections. In contrast, chemotherapy status showed no significant association with CRKP infection (*p* = 0.674) (Table 7).

**Table 7:** Association of clinical history and treatment status with CRKP infection

Clinical / treatment variables	CRKP (*N* = 129)	Non-CRKP (*N* = 112)	*p* value
**Prior antibiotic use**	**n (%)**	**n (%)**	
Yes	78 (60.5)	47 (42)	0.0041
No	51 (39.5)	65 (58)	
**Hospitalization (> 3days)**			
Yes	74 (57.4)	19 (17)	< 0.001
No	55 (42.6)	93 (83)	
**ICU admission**			
Yes	21 (16.3)	7 (6.3)	0.015
No	108 (83.7)	105 (93.8)	
**Chemotherapy status**			
On treatment	106 (82.2)	81 (72.3)	0.674
Not on treatment	23 (17.8)	31 (27.7)	
**Fever**			
Yes	96 (74.4)	68 (60.7)	0.0228
No	33 (25.6)	44 (39.3)	

## Discussion

Carbapenem-resistant *Klebsiella pneumoniae* (CRKP) and hypervirulent *K. pneumoniae* (hvKP) are increasingly recognized as major clinical threats, particularly among immunocompromised populations such as cancer patients. However, data from Nepal regarding their molecular characteristics and coexistence remain limited. This study provides important insights into the prevalence, antimicrobial resistance patterns, and genetic determinants of *K. pneumoniae* in this high-risk group.

The prevalence of *K. pneumoniae* (23.7%) observed in this study is comparable to findings from Nepal and neighboring regions [30, 31], although variations exist across studies [32, 33]. Such differences may reflect heterogeneity in study populations, diagnostic approaches, and infection control practices. The relatively higher prevalence in this study may be attributed to the immunocompromised status of cancer patients, who are more susceptible to opportunistic infections due to chemotherapy and repeated healthcare exposure.

A higher infection rate was observed among males and elderly patients (≥ 61 years). These findings are consistent with previous reports [34] and may be explained by sex-related immunological differences, comorbid conditions, and age-associated immune decline.

The high proportion of prior antibiotic use (52%) observed in this study supports its established role as a key driver in the emergence of multidrug-resistant organisms through repeated or prolonged exposure to antimicrobials [35]. Fever, present in 68% of cases, was the most common clinical feature, likely attributable to chemotherapy-induced neutropenia and impaired immune responses; in such patients, fever often serves as the earliest indicator of underlying bacterial infection [36].

The majority of patients were undergoing chemotherapy (77.6%), reflecting significant immunosuppression that increases susceptibility to infections and facilitates colonization by resistant organisms [37]. Additionally, hospitalization exceeding 3 days (38.5%) and ICU admission (11.6%) highlight the contribution of healthcare-associated factors. Prolonged hospital stay increases exposure to invasive procedures and contaminated environments, while ICU settings, characterized by critical illness and intensive antibiotic use, further promote antimicrobial resistance [38].

The predominance of urinary isolates aligns with earlier studies, indicating that urinary tract infections remain a common clinical manifestation of *K. pneumoniae* in hospitalized patients [39].

The antimicrobial susceptibility profile demonstrated a concerning level of resistance, with almost complete resistance to amoxicillin-clavulanic acid (99.6%) and relatively low resistance to tigecycline (9.5%). These findings are in agreement with previous studies [29, 30]. The observed susceptibility to colistin suggests its continued efficacy; however, its use should be cautious due to potential toxicity and the risk of emerging resistance. These results highlight the urgent need for antimicrobial stewardship and rational antibiotic use.

Molecular analysis revealed that 53.5% of isolates harbored carbapenemase genes, with *bla*_NDM−1_ being the most prevalent. This is consistent with studies conducted in Nepal [40, 41] and in Egypt [42], supporting the global dissemination of *bla*_NDM_ type enzymes. The predominance of *bla*_NDM−1_ may be attributed to its association with mobile genetic elements that facilitate horizontal gene transfer [43, 44]. Notably, a substantial proportion of isolates lacked the tested carbapenemase genes, suggesting the involvement of alternative resistance mechanisms, such as porin loss, efflux pump overexpression, or the presence of uncharacterized genes [45]. The absence of *bla*_OXA−48–like_ genes may be explained by their relatively weak intrinsic carbapenemase activity, which often requires additional mechanisms such as porin loss for full resistance expression, while the absence of *bla*_KPC_ may reflect limited regional dissemination and reduced clonal or plasmid-mediated spread of KPC-producing strains in the present setting [46].

A significant proportion of CRKP isolates carried hypervirulence-associated genes, with iucA being the most frequently detected. This finding is consistent with previous studies [47–49] and highlights the role of aerobactin-mediated iron acquisition in bacterial pathogenicity. Other virulence genes, including *iroB*,* rmpA*,* rmpA2*, and *peg-344*, were detected at lower frequencies, in agreement with earlier reports [27]. Interestingly, only 36.4% of isolates were positive in the string test, indicating that the presence of virulence genes does not necessarily correlate with phenotypic expression. This discrepancy may be due to regulatory mechanisms, environmental influences, or genetic variability.

The coexistence of carbapenem resistance and hypervirulence is of particular concern. In this study, a significant association was observed between carbapenemase genes and virulence determinants, supporting previous evidence of convergence between resistance and virulence traits [50–52]. The detection of carbapenem-resistant hypervirulent *K. pneumoniae* (CR-hvKP) in 21% of isolates underscores the emergence of highly pathogenic strains with limited therapeutic options and increased clinical severity [53–55]. This convergence is likely driven by horizontal gene transfer or clonal expansion and poses a substantial public health threat.

Analysis of clinical risk factors revealed that prior antibiotic use, prolonged hospitalization, ICU admission, and fever were significantly associated with CRKP infection. Carbapenem-resistant *K. pneumoniae* (CRKP) isolates were significantly more frequent among patients with prolonged hospitalization compared to non-CRKP isolates, indicating a possible association with hospital-acquired infections. Prolonged hospitalization appears to be a significant risk factors, it should be interpreted as part of a broader multifactorial framework rather than an independent indicator of hospital acquired infections [56]. These findings emphasize the role of healthcare-associated exposure and antimicrobial pressure in the selection of resistant strains. In contrast, chemotherapy was not significantly associated, suggesting that environmental and clinical factors may play a more critical role than treatment status alone.

This study has some limitations. It was conducted at a single center limiting generalizability. Only selected resistance and virulence genes were analyzed. KPC phenotypic detection was not performed, which may underestimate certain carbapenemase producers. The string test was performed only on CRKP isolates, potentially overlooking hypervirulent strains among non-CRKP isolates. Additionally, clinical outcome data were not included, and the cross-sectional design limits assessment of transmission dynamics and temporal trends.

## Conclusion

In conclusion, this study highlights the increasing burden of CRKP and the concerning convergence of resistance and hypervirulence in *K. pneumoniae* isolates from cancer patients. The findings underscore the need for continuous molecular surveillance, implementation of robust infection control strategies, and strengthening of antimicrobial stewardship programs. Further genomic studies are warranted to better understand the mechanisms driving the coexistence of resistance and virulence and their clinical implications.

## Supplementary Information

Below is the link to the electronic supplementary material.

Supplementary Material 1

Supplementary Material 2

## Data Availability

The datasets generated and/or analyzed during the current study are available from the corresponding author on reasonable request.

## Abbreviations

*bla*_*NDM*_: New Delhi metallo–β–lactamase
*bla*_*VIM*_: Verona integron–encoded metallo–β–lactamase
*bla*_*IM*P_: Imipenemase metallo–β–lactamase
*bla*_*KPC*_: *Klebsiella pneumoniae* carbapenemase
*bla*_*OXA−48*_: Oxacillinase–48
CRKP: Carbapenem resistant *Klebsiella pneumoniae*
CRhvKP: Carbapenem resistant hypervirulent *Klebsiella pneumoniae*
DNA: Deoxyribonucleic Acid
EDTA: Ethylenediamine Tetra acetic Acid
hvKP: Hypervirulent *Klebsiella pneumoniae*
ICU: Intensive Care Unit
*iucA*: Aerobactin siderophore system
*iroB*: Salmochelin siderophore system
MBL: Metallo- β-lactamase
MDR: Multi-drug Resistant
*peg344*: Putative metabolic transporter, hvKp marker
PCR: Polymerase Chain Reaction
*rmpA*: Regulator of mucoid phenotype A
*rmpA2*: Regulator of mucoid phenotype A2
WHO: World Health Organization
