# Supplementary material for: Detection of hypervirulence genes in carbapenem resistant Klebsiella pneumoniae from cancer patients at a tertiary referral hospital in Nepal
Source: BMC Infect Dis. 2026 Apr 23;26:1101. doi: 10.1186/s12879-026-13382-8 (PMC13255225; doi:10.1186/s12879-026-13382-8)
Supplement: Supplementary file 1 — Supplementary Material 1 [file 12879_2026_13382_MOESM1_ESM.docx]

**Supplementary Table 1:** The sequence of oligonucleotide primers used for PCR amplification of the carbapenemase-encoding genes and hyper-virulence genes

| **Carbapenemase-encoding genes** | |  |  |  |  |
| --- | --- | --- | --- | --- | --- |
| Gene | Target & Sequence (5’ to 3’) | Amplicon(bp) | Thermal cycles | Reference | |
| blaVIM-2 | F: TTTGGTCGCATATCGCAACG | 390 bp | 94 °C, 5 mins, 35 cycles, 94 °C 45 secs, 55 °C 45secs, 72 °C 45 secs ,72 °C 7mins | Ranjbar R.Farahani 2019 | |
|  | R: CCATTCAGCCAGATCGGCAT |  |  |  |  |
|  |  |  |  |  |  |
|  | | | | | |
| blaIMP-1 | F:GGAATAGAGTGGCTTAAYTCTC R: GGTTTAAYAAAACAACCACC | 234bp | 94 °C, 5 mins, 35 cycles, 94 °C 45 secs, 55 °C 45secs, 72 °C 45 secs ,72 °C 7mins | Hatrangjit R et al 2018 | |
|  | | | | | |
| bla NDM-1 | F : GGTTTGGCGATCTGGTTTTC R: CGGAATGGCTCATCACGATC | 621bp | 94 °C, 5 mins, 35 cycles, 94 °C 45 secs, 52 °C 45secs, 72 °C 45 secs ,72 °C 7mins | Poirel, et al.,2011) | |
|  | | | | | |
| **bla KPC** | **F:GTATCGCCGTCTAGTTCTGC R:GGTCGTGTTTCCCTTTAGCC** | **368** | **94 °C, 5 mins, 35 cycles, 94 °C 45 secs, 56 °C 45secs, 72 °C 45 secs ,72 °C 7mins** | Russo et al., 2018 | |
|  | | | | | |
| **bla OXA-48** | F:TTGGTGGCATCGATTATCGG | **743** | **94 °C, 5 mins, 35 cycles, 94 °C 45 secs, 62 °C 45secs, 72 °C 45 secs ,72 °C 7mins** | Russo et al., 2018 | |
|  | R:GAGCACTTCTTTTGTGATGGC |  |  |  |  |
| **Hypervirulennce-encoding genes** | | | | | |
| Gene | Target & Sequence (5’ to 3’) | Amplicon(bp) | | Reference | |
| IucA | F:-GCT GGT GGT TTA CCG GAA A R: GCG TCG ATG TTA CGA TGT TG | 239bp | 94 °C, 10 mins, 35 cycles, 94 °C 40 secs, 60 °C 40secs, 72 °C 40 secs ,72 °C 5 mins | Russo et al., 2018 | |
|  | | | | | |
| IroB | F: ATG GCT ACG GCA AAC GAA -3′ | 498bp | 94 °C, 10 mins, 35 cycles, 94 °C 40 secs, 60 °C 40secs, 72 °C 40 secs ,72 °C 5 mins | Russo et al., 2018 | |
|  | R:TTC AGC ATC GCG TGA ATC |  |  |  |  |
|  | | | | | |
| rmpA | F:ACT GGG CTG ATA GAG GCA TA | 516bp | 94 °C, 10 mins, 35 cycles, 94 °C 40 secs, | Russo et al., 2018 |  |
|  | R: GGC CAT GCT GGA AAC ATA AT | | 53 °C 40secs, 72 °C 40 secs ,72 °C 5 mins | |  |
|  |  |  |  |  |  |
| rmpA2 | F:CGC GAT GCT GTT TTC ATT GA | 450bp | 94 °C, 10 mins, 35 cycles, 94 °C 40 secs, | Russo et al., 2018 |  |
|  | R:TTC GAT GGT GGT GTT GTT GT |  | 55 °C 40secs, 72 °C 40 secs ,72 °C 5 mins | |  |
|  |  |  |  |  |  |
| peg344 | F:GGT TGG TAA CCG CTA TTG CT | 683bp | 94 °C, 10 mins, 35 cycles, 94 °C 40 secs, | Russo et al., 2018 |  |
|  | R:TGA CCA GCA TTT CCA CTT GA |  | 50°C 40secs, 72 °C 40 secs ,72 °C 5 mins | |  |
