# Supplementary material for: Detection of hypervirulence genes in carbapenem resistant Klebsiella pneumoniae from cancer patients at a tertiary referral hospital in Nepal
Source: BMC Infect Dis. 2026 Apr 23;26:1101. doi: 10.1186/s12879-026-13382-8 (PMC13255225; doi:10.1186/s12879-026-13382-8)
Supplement: Supplementary file 2 — Supplementary Material 2 [file 12879_2026_13382_MOESM2_ESM.docx]

Supplementary Table 2: Distribution of carbapenemase and hypervirulence encoding gene–combination patterns among CRKP isolates (N = 129)

| **SN** | **Gene combination pattern** | **n (%)** |
| --- | --- | --- |
| 1 | bla_NDM1_ only | 25 (19.4) |
| 2 | iucA + rmpA2 | 18 (14.0) |
| 3 | iucA only | 15 (11.6) |
| 4 | bla_NDM1_ + iucA | 15 (11.6) |
| 5 | iucA + iroB + rmpA2 | 14 (11) |
| 6 | No gene detected | 4 (3) |
| 7 | iucA + iroB | 10 (7.8) |
| 8 | bla_NDM1_ + rmpA2 | 2 (1.5) |
| 9 | bla_NDM1_ + iucA + iroB | 1 (0.8) |
| 10 | iucA + iroB + peg344 + rmpA + rmpA2 | 3 (2.3) |
| 11 | iucA + peg344 + rmpA2 | 6 (4.6) |
| 12 | bla_VIM2_ only | 1(0.8) |
| 13 | bla_IMP1_ only | 1 (0.8) |
| 14 | iucA + peg344 | 2 (1.5) |
| 15 | peg344 only | 1 (0.8) |
| 16 | bla_VIM2_ + peg344 | 1 (0.8) |
| 17 | bla_IMP1_ + iucA | 1 (0.8) |
| 18 | bla_VIM2_ + iucA | 1 (0.8) |
| 19 | iroB only | 1 (0.8) |
| 20 | rmpA2 only | 1 (0.8) |
| 21 | bla_NDM1_ + iroB | 6 (4.6) |
|  | **Total** | **129 (100)** |
